# Fluoroquinolone resistance during 2000–2005 : An observational study

**DOI:** 10.1186/1471-2334-8-71

**Published:** 2008-05-24

**Authors:** Richard J Ryan, Chris Lindsell, Paul Sheehan

**Affiliations:** 1 Department of Emergency Medicine, University of Cincinnati, 231 Albert Sabin Way, Cincinnati, Ohio 45267-0769, USA

## Abstract

**Background:**

Moxifloxacin is a respiratory fluoroquinolone with a community acquired pneumonia indication. Unlike other fluoroquinolones used in our healthcare system, moxifloxacin's urinary excretion is low and thus we hypothesized that increased use of moxifloxacin is associated with an increase in fluoroquinolone resistance amongst gram negative uropathogens.

**Methods:**

All antibiograms for Gram negative bacteria were obtained for 2000 to 2005. The defined daily dose (DDD) for each fluoroquinolone was computed according to World Health Organization criteria. To account for fluctuation in patient volume, DDD/1000 bed days was computed for each year of study. Association between DDD/1000 bed days for each fluoroquinolone and the susceptibility of Gram negative bacteria to ciprofloxacin was assessed using Pearson's Correlation Coefficient, r.

**Results:**

During the study period, there were 48,261 antibiograms, 347,931 DDD of fluoroquinolones, and 1,943,338 bed days. Use of fluoroquinolones among inpatients decreased from 237.2 DDD/1000 bed days in 2000 to 115.2 DDD/1000 bed days in 2005. With the exception of *Enterobacter aerogenes*, moxifloxacin use was negatively correlated with sensitivity among all 13 Gram negative species evaluated (r = -0.07 to -0.97). When the sensitivities of all Gram negative organisms were aggregated, all fluoroquinolones except moxifloxacin were associated with increased sensitivity (r = 0.486 to 1.000) while moxifloxacin was associated with decreased sensitivity (r = -0.464).

**Conclusion:**

Moxifloxacin, while indicated for empiric treatment of community acquired pneumonia, may have important negative influence on local antibiotic sensitivities amongst Gram negative organisms. This effect was not shared by other commonly used members of the fluoroquinolone class.

## Background

Antibiotic resistance places a large burden on the healthcare system, with both increased costs and increased morbidity and mortality. Infection with resistant bacteria can double hospital length of stay and associated costs [1]. As the problem of antibiotic resistance has grown, awareness of induction of resistance from antibiotic use has increased. This concept has most firmly taken root in the Intensive Care Unit (ICU), where empiric antibiotic regimens have been developed not only for safety and efficacy of treatment, but also to prevent the rise of resistant organisms [2].

The traditional antimicrobial therapy of choice in any circumstance has been a regimen that is efficacious, safe, and inexpensive. However, if that regimen induces antibiotic resistance then efficacy soon suffers. Moxifloxacin is a respiratory fluoroquinolone with an indication for community acquired pneumonia (CAP). Moxifloxacin's broad spectrum coverage has also gained the drug indications for acute bacterial exacerbations of chronic bronchitis, acute maxillary sinusitis, skin and soft tissue infections, and more recently complicated intra-abdominal infections. However, compared to other commonly used fluoroquinolones, only 20% of moxifloxacin is excreted into the urinary tract [3]. We hypothesized that increased use of moxifloxacin is associated with an increase in fluoroquinolone resistance amongst Gram negative organisms.

## Methods

This observational study used antibiogram data from a three hospital healthcare system sharing the same drug formulary. The quantity and volume of doses of fluoroquinolone given over a six year period (2000 to 2005) were provided by the hospital pharmacy system, and the number of inpatient hospital bed days were extracted from central databases. Only deidentified summary data were provided, and thus this study was exempt from Institutional Review Board oversight.

The three hospitals include an urban, tertiary care academic center with 662 beds, an urban community hospital with 555 beds, and a suburban community hospital with 200 beds. The academic center treats the majority of the region's indigent patient population under a tax levy system. The three hospitals primarily admit adults; those under the age of 18 are usually treated at the region's children's hospital.

Antibiograms are generated by the core laboratory from inpatient blood cultures, and reported in aggregate on a yearly basis. Susceptibility to ciprofloxacin is the system's preferred marker for susceptibility to fluoroquinolones. Susceptibility to ciprofloxacin was determined by minimum inhibitory concentration (MIC) with an MIC of <1 microgram/ml considered susceptible. All antibiograms for blood cultures growing Gram negative bacteria were obtained for each year from 2000 to 2005. Four main antibiotics in the fluoroquinolone class were used during the study period: ciprofloxacin, levofloxacin, gatifloxacin, and moxifloxacin. Gatifloxacin was removed from use during 2002 secondary to concerns about the side-effect profile, and was replaced with other respiratory fluoroquinolones.

The defined daily dose (DDD) for each antibiotic was computed according to World Health Organization criteria to account for variations in IV and PO dosing [4]. To account for fluctuation in patient volume, DDD/1000 bed days was computed for each year of study. Association between DDD/1000 bed days for each FQ and the susceptibility of Gram negative bacteria to ciprofloxacin was assessed using Pearson's Correlation Coefficient.

## Results

**Figure 1:**
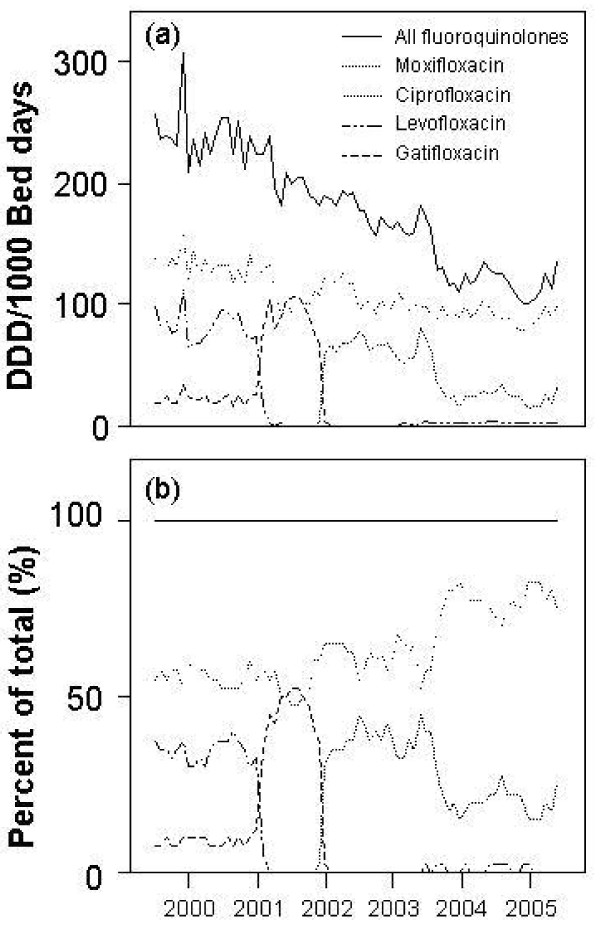
**Defined daily doses for each fluoroquinolone, and for all fluoroquinolones adjusted for patient volume for each month of the study (Panel A).** The percentage contribution of each fluoroquinolone to the total is also shown (Panel B). Year midpoints are labeled.

During the study period, there were 1,943,338 inpatient bed days. A total of 48,261 antibiograms for gram negative bacteria were generated, and 347,931 DDD of fluoroquinolones were given. [Additional file 1] shows the number of susceptible isolates and total isolates for Gram negative bacteria for each year of the study. Use of fluoroquinolones among inpatients decreased from 237.2 DDD/1000 bed days in 2000 to 115.2 DDD/1000 bed days in 2005 (Figure 1). Moxifloxacin use increased during the period of study, whereas all other fluoroquinolones showed a decrease in usage. Moxifloxacin use peaked in 2003–2004, after which the proportion of moxifloxacin to total fluoroquinolone usage remained approximately constant.

Table 1 shows the correlations between antibiotic usage and sensitivity to fluoroquinolones. Sample size was 6 (n = 1 for each year of study), thus a correlation coefficient above 0.81 is significant at the 5% level. With the exception of *Enterobacter aerogenes*, moxifloxacin use was negatively correlated with sensitivity among all Gram negative species evaluated. When the sensitivities of Gram negative organisms were aggregated, all fluoroquinolones except moxifloxacin were associated with increased sensitivity, while moxifloxacin was associated with decreased sensitivity (r = -0.636).

**Table 1:** Correlation between DDD/1000 bed days and sensitivity to gram negative bacteria by antibiogram (N = 6, years 2000–2005)

**Organism**	**DDD/1000 bed days**				
	**Ciprofloxacin**	**Levofloxacin**	**Gatifloxacin**	**Moxifloxacin**	**All**
*Acinetobacter baumanii complex*	0.682	0.410	0.681	**-0.158**	0.775
*Citrobacter freundii complex*	0.856	0.706	0.482	**-0.238**	0.906
*Citrobacter koseri*	0.511	0.645	0.505	**-0.970**	0.394
*Enterobacter aerogenes*	-0.559	-0.671	-0.627	**0.887**	-0.529
*Enterobacter cloacae*	0.956	0.840	0.789	**-0.654**	0.978
*Escherichia coli*	0.994	0.873	0.745	**-0.666**	0.987
*Klebsiella oxytoca*, Non-ESBL	0.649	0.388	0.509	**-0.067**	0.709
*Klebsiella pneumoniae*, Non-ESBL	0.457	0.269	0.364	**-0.162**	0.442
*Morganella morganii*	0.769	0.776	0.643	**-0.921**	0.666
*Proteus mirabilis*	0.996	0.878	0.651	**-0.587**	0.984
*Providencia stuartii*	0.780	0.605	0.726	**-0.350**	0.872
*Pseudomonas aeruginosa*	-0.119	0.320	-0.417	**-0.522**	-0.278
*Serratia marcescens*	0.665	0.584	0.436	**-0.602**	0.553

All organisms	0.995	0.890	0.670	**-0.636**	0.978

## Discussion

Our study results show an association between the use of moxifloxacin in our healthcare system and an increase in fluoroquinolone resistance amongst Gram negative bacteria. The efficacy of moxifloxacin against *S. pneumoniae *has been documented in multiple studies across multiple patient populations [5-7]. Additionally, moxifloxacin has documented *in vivo *efficacy against Gram negative bacteria a variety of body tissues [8,9]. In contrast, moxifloxacin is not FDA approved for the treatment of urinary tract infections, and no published clinical data exists on moxifloxacin's efficacy in urinary tract infections.

Fluoroquinolones exhibit concentration dependent bactericidal effects, and urine excretion of moxifloxacin is half that of the next closest fluroquinolone used in our healthcare system (ciprofloxacin at 43% [3]). Urine concentrations after a single dose of moxifloxacin in healthy volunteers have previously been found to be effective *in vitro *against levofloxacin susceptible and moderately susceptible strains of *E. Coli, E. faecalis, and K. pneumoniae *but not *P. aeruginosa *[10]. However, the relatively low excretion of moxifloxacin into the urine is particularly important due to the rising North American MIC_90 _of moxifloxacin against *E. Coli*. The TRUST 11 surveillance database demonstrated an MIC_90 _of 32 mcg/ml for moxifloxacin against *E. Coli*.

In addition, *in vivo *urine bactericidal concentrations may be much higher then *in vitro *due to biofilm [3] and pH effects [10]. Biofilms have been demonstrated to markedly increase needed minimum inhibitory concentrations (MICs) and a recent study by Rosen *et al *found filamentous bacteria were common even in healthy women with uncomplicated cystitis [11]. Additionally, fluoroquinolones have been shown to have be less active in acidic urine (pH 5) against *E. Coli *than in a broth medium [12]. As fluoroquinolone activity is concentration dependent, these factors may contribute to a differential in a sub-therapeutic exposure among urinary pathogens between moxifloxacin and other fluoroquinolones that are excreted in higher urinary concentrations. Sub-therapeutic exposure may result in selection of fluoroquinolone resistant among Gram negative bacteria dwelling in the urinary tract through previously established resistance mechanisms, such as mutations in DNA gyrase or development of efflux pumps [13].

An alternative mechanism for increasing resistance among Gram negative organisms was discussed by von Baum *et al*. [14] Their study found a doubling in the incidence of Gram negative bacteremia among patients given moxifloxacin as prophylaxis during neutropenia compared to historical controls given levofloxacin. It was proposed that this effect was due to moxifloxacin's superior efficacy against anaerobes. Elimination of native intestinal flora causesa loss of intestinal colonization resistance which may have favored colonization with fluoroquinolone resistant *E. coli*. Joris *et al*. [15] was able to show an increase in fluoroquinolone resistant Gram negative bacilli effect in healthy volunteers exposed to ciprofloxacin in combination with a drug with good efficacy against anaerobes, clindamycin. This increase was not seen in patients on ciprofloxacin alone.

In our health care system, ciprofloxacin is still a primary treatment for urinary tract infections. The increasing local resistance of Gram negative bacteria to ciprofloxacin has important consequences for empiric treatment of urosepsis and other Gram negative bacteremias. Further investigations should include microbiological investigation of the proposed mechanism of increasing resistance, as well as assessing the effect of outpatient fluoroquinolone prescriptions on resistance within the healthcare system.

### Limitations

While our findings demonstrate an association between increased use of moxifloxacin and sensitivity of gram negative bacteria to fluoroquinolone, this was a retrospective review of antibiotic usage and microbial sensitivity. This study design is incapable of establishing a causal link between moxifloxacin usage and increasing fluoroquinolone resistance. Also, it is currently thought that fluoroquinolone resistance is not significantly influenced by other antimicrobial agents, and thus analysis was limited to the fluoroquinolone class. No formal microbiological studies were undertaken to demonstrate this property within our healthcare system. Finally, this study only considered inpatient antibiotic usage and inpatient blood cultures. It is possible that increases in non-moxifloxacin fluoroquinolone prescriptions to outpatients during the study period may be confounding the observed relationships.

## Conclusion

Moxifloxacin, while indicated for empiric treatment of respiratory, soft tissue, and intra-abdominal infections, is associated with a negative influence on local antibiotic sensitivities amongst Gram negative organisms. This association does not seem to be shared by other commonly used members of the fluoroquinolone class used by our healthcare system. Further studies are needed to determine if there is a causal link between moxifloxacin use and Gram negative resistance to fluoroquinolones, as well as the exact mechanism of this increasing resistance.

## Competing interests

The authors declare that they have no competing interests.

## Authors' contributions

RJR: developed the initial study question, as well as acquisition of preliminary data and assistance with study design, manuscript preparation and overall direction. PS: literature search, data collection, and manuscript preparation. CL: biostatistical expertise, assisted with both study design and manuscript preparation.

## Pre-publication history

The pre-publication history for this paper can be accessed here:



## Supplementary Material

Additional file 1 Sensitivity of Gram negative organisms to Ciprofloxacin by year. The total number of isolates (n) and percent sensitivity (%) is given. Click here for file
