# Supplementary figures and images for: Fluoroquinolone resistance during 2000–2005 : An observational study
Source: BMC Infect Dis. 2008 May 24;8:71. doi: 10.1186/1471-2334-8-71 (PMC2424048; doi:10.1186/1471-2334-8-71)

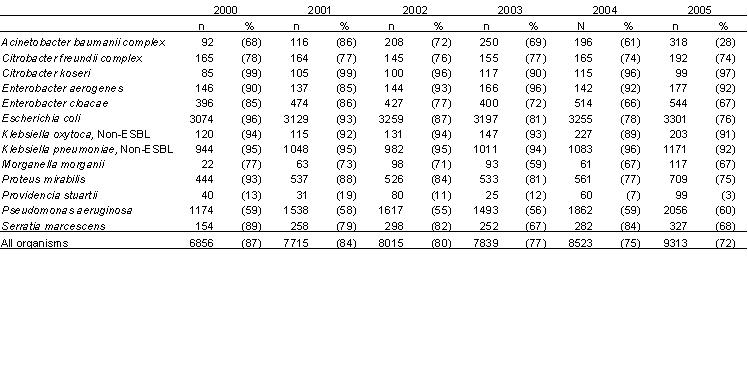

Supplement: Additional file 1 — Sensitivity of Gram negative organisms to Ciprofloxacin by year. The total number of isolates (n) and percent sensitivity (%) is given. [file 1471-2334-8-71-S1.jpeg]
